# The sport persistence training dataset: a randomized controlled pre–post dataset on sport persistence and related psychological resources among young athletes in Hungary

**DOI:** 10.3389/fpsyg.2026.1921608

**Published:** 2026-10-08

**Authors:** Karolina Eszter Kovács

**Affiliations:** 1 Institute of Psychology, Faculty of Arts, University of Debrecen, Debrecen, Hungary; 2 Department of Psychology and Sport Psychology, Hungarian University of Sports Science, Budapest, Hungary

**Keywords:** Hungary, intervention, pre-post testing, sport persistence, sport training

## 1 Introduction

Sport participation is widely recognized as an important contributor to physical health, psychological wellbeing, and social development (Gyömbér and Kovács, 2012). However, maintaining long-term engagement in sport remains challenging, with participation differing by gender (Hanrahan and Cerin, 2009; Pikó and Keresztes, 2007) and typically declining across adolescence and early adulthood (Ingrell et al., 2020; Németh, 2023). These trends underline the need to better understand the factors that promote sustained engagement in sport.

Sport persistence represents a complex psychological and behavioral construct that extends beyond the frequency of participation and reflects a qualitative commitment to long-term sporting activity (Kovács, 2021). It encompasses athletes' capacity to maintain effort despite setbacks such as injuries, performance plateaus, failures, or other challenges, while also effectively utilizing positive experiences to sustain development. Consequently, sport persistence can be considered an indicator of effective and enduring sport participation rather than mere attendance. Despite its theoretical importance, empirical research has primarily focused on sport motivation, commitment, or participation, leaving sport persistence relatively underexplored (Kovács et al., 2022).

Previous studies have also demonstrated consistent gender differences in sport persistence (Kovács, 2025a,b; Tóth et al., 2026). These differences are likely shaped by socialization processes, gender-specific expectations, and variations in motivational development. Boys and girls may internalize achievement goals and competitive experiences differently, potentially influencing both grit and sport orientation (Grugan et al., 2021). Consequently, these psychological constructs may partially explain how gender differences emerge in long-term commitment to sport.

Although the determinants of sport motivation and commitment have been widely investigated, relatively little attention has been devoted to sport persistence as a distinct construct, and evidence-based interventions specifically targeting its development remain scarce. Building on our previous research (Kovács, 2025a,b; Kovács and Csukonyi, 2025; Selejó Joó et al., 2024; Tóth et al., 2024), the present study contributes to this emerging field by examining sport persistence within a comprehensive psychological framework and evaluating an intervention designed to strengthen athletes' long-term commitment to sport.

The dataset is particularly relevant because it goes beyond one focal outcome. In addition to sport persistence, it includes measures that can support secondary analyses of motivational regulation, anxiety, coping-related functioning, subjective wellbeing, future orientation, and repetitive negative thinking. This breadth makes the dataset suitable not only for intervention evaluation but also for psychometric studies, cross-construct association testing, subgroup comparisons, attrition analyses, and methodological work on multi-scale athlete datasets. The present article therefore does not report the findings of a single hypothesis-driven study; instead, it documents the origin, structure, quality indicators, and reuse potential of the dataset.

The intervention context is also an important part of the dataset. The sport persistence training programme was developed as a multidimensional psychological workbook and delivery framework intended to support long-term commitment, mental resilience, and performance-related self-regulation. The intervention materials cover self-assessment, anxiety and coping, self-management and time management, communication, attachment, and social support. Because these materials are explicitly linked to the data collection design, they provide important context for interpreting change scores, format-specific exposure, and the practical meaning of the variables captured in the dataset.

## 2 Methods

### 2.1 Study design

The dataset was generated in a randomized controlled trial involving athletes aged 14–25 years in Hungary. Recruitment was carried out between September 2025 and November 2025, the intervention period ran from October to December 2025, and post-intervention follow-up assessments were completed between November 2025 and January 2026. Eligibility screening initially covered 300 individuals. The design used a pre-test/post-test questionnaire battery and three study conditions: a hybrid training format, an online self-administered training format, and a no-training control group. This timeline and structure should be reported explicitly because they determine how the repository files can be interpreted for longitudinal, between-group, and format-sensitive reuse.

Participants were recruited through sports clubs and secondary schools. Inclusion criteria were clearly defined: participants had to be 14–25 years old, engage in sport at least three times per week, practice either an individual or team sport, and be enrolled in secondary or higher education. Exclusion rules applied where valid completion of the measures or intervention was not possible, where baseline or post-intervention participation was missing, where primary questionnaire data were incomplete, or where parental/guardian permission was not available for minors under the approved protocol. For dataset users, these criteria are important because they define the analytic population and limit generalization primarily to actively training student-athletes rather than the broader youth population.

Random allocation followed a simple computer-generated procedure with a 2:1 intervention-to-control ratio. Participants were randomly allocated using a computer-generated randomization sequence. Participants were informed of their allocation after randomization because delivery of the intervention differed between study arms. After eligibility screening, 281 participants were randomized. Of these, 188 were assigned to the intervention arm and 93 to the control arm. Within the intervention arm, 86 participants entered the hybrid format and 102 the online format. Follow-up attrition occurred in both main arms, mainly because post-intervention tasks were not completed. The final analysis-ready sample consisted of 208 complete cases: 139 in the intervention arm, including 60 hybrid and 79 online participants, and 69 in the control arm. Because the dataset supports both intention-sensitive design description and completer-based secondary analysis, the repository documentation should clearly distinguish screened, randomized, allocated, follow-up, and analysis-ready cases (Figure 1).

**Figure 1:**
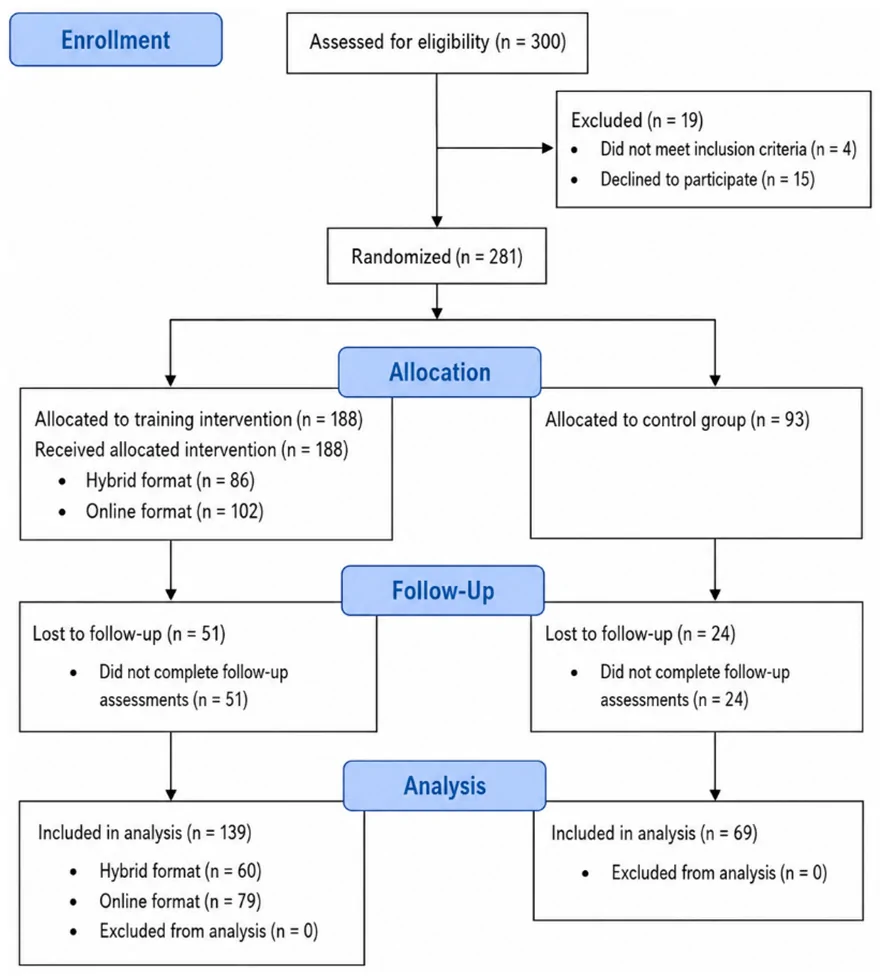
Sample composition based on the CONSORT diagram (Hopewell et al., 2025).

Anonymization relied on participant-generated codes that linked repeated measurements without storing identifiers in the research database itself. According to the study documentation, the separate file used for verification purposes was stored independently, password-protected, and accessible only to the principal investigator. For a public dataset release, only the de-identified analytical files and metadata are openly available. The OSF repository includes the anonymized dataset, a complete codebook, dataset documentation, and the intervention workbook materials. The codebook describes the variable names, coding schemes, participant identifiers, measurement waves, group allocation variables, missing-value coding, and scoring procedures required for data reuse.

### 2.2 Procedure

The intervention materials are an essential interpretive layer of the dataset. The workbook is organized into six blocks: self-assessment; anxiety, resilience, and wellbeing; self-management and time management; communication skills; attachment; and social support. The workbook also contains practical exercises such as success journaling, reframing negative automatic thoughts, Pomodoro-based time structuring, tactical communication signals, motivational keyword mapping, and social support network mapping. In addition, the usage instructions encourage stepwise completion, journaling, collaboration with coaches or teammates where relevant, and repeated revisiting of the material. These features matter because the dataset does not merely record exposure to an intervention label; it records participation in a structured psychological training environment with recognizable content domains (Table 1).

**Table 1:** The modules of the sport persistence training programme, their objectives and sample tasks.

Block	Objective	Sample task
Block 1: self-assessment	To help athletes become aware of their areas for improvement and limitations, while also recognizing their personal values.	Success Journal: write down 2–3 important sporting achievements and note which of your strengths (e.g., perseverance, discipline) helped you accomplish them. Then reread them to remind yourself of what you are good at.
Block 2: anxiety, resilience, and wellbeing	To help athletes understand the role these three components play in sporting success and to provide them with tools for overcoming anxiety.	Write down the negative automatic thoughts that contribute to your anxiety, then rephrase them so that they no longer feel threatening.
Block 3: self-management and time management	Athletes face diverse daily demands; therefore, balancing training, competitions, studies or work, and recovery requires conscious planning. Self-management supports the maintenance of discipline and motivation, while effective time management reduces the risk of burnout and performance fluctuations caused by overload.	Try the Pomodoro Technique during a study session: work with full focus for 25 min, then take a 5-min break. Observe how much more effectively you work using this method.
Block 4: development of communication skills	Effective, open, and assertive communication fundamentally influences the athlete–coach relationship, team cohesion, and performance, while also helping to prevent and manage conflicts.	Create shared tactical signals (e.g., hand gestures), practice using them during training, and observe how much easier communication becomes.
Block 5: attachment	To foster healthy bonds with both the athlete's social environment and sporting activity.	Write down keywords that describe you as an athlete and motivate you. Then identify which words appear most frequently—these reflect your strengths and your motivation toward sport.
Block 6: social support	An athletic career is full of challenges, during which adequate social support plays a crucial role in maintaining psychological wellbeing and commitment.	List the people who make up your support network and note what kind of help you receive from each of them. This will help you see who you can rely on and for what type of support.

The questionnaire package covered socio-demographic, sport-specific, health-related, and psychological domains. The comprehensive study also utilized additional measurement tools that are not the subject of this study: a sports- and health-specific questionnaire, the perception of success questionnaire (PSQ, Roberts et al., 1998), the Sport Anxiety Scale-2 (SAS-2, Smith et al., 2006), the Connor-Davidson Resilience Scale (Járai et al., 2015), the WHO wellbeing inventory (WBI-5, Susánszky et al., 2006), Self in the Future Scale (Dombi, 2018), and the Ruminative Response Scale (RRS, Kocsel et al., 2019).

The dataset should be accompanied by clear variable-level documentation. At minimum, the repository package should identify each scale, its item count, response format, reverse-coded items where applicable, subscale composition, score range, and whether a variable belongs to baseline, post-intervention, or derived difference-score files. If multiple files are deposited, it is advisable to separate raw de-identified item-level data, scored scale-level data, and documentation files. Because Frontiers requires datasets to be reusable and interpretable by readers beyond the original study team, the documentation of scoring decisions and filters is not optional background information but a central part of the publication.

## 3 Descriptive overview and basic analysis of the data

The descriptive profile of the complete-case sample indicates broad heterogeneity within an active youth athlete population. Among the 208 analyzed participants, the gender distribution was relatively balanced, with 112 males and 96 females. Most participants were enrolled in secondary education, and a smaller but substantial subgroup was in higher education. The sample included athletes from a range of settlement types, although county towns and small towns were most common. In sport participation terms, the sample was highly active: most trained several times a week or more often, most were involved in team sports, many were linked to sports clubs, and the majority identified as competitive rather than purely recreational athletes. A notable proportion also reported prior sports injury, which increases the potential for secondary analyses on injury history and persistence-related functioning.

The participant-flow information is itself analytically useful. Screening, exclusion, randomization, intervention-format allocation, and follow-up loss were all documented. This means the dataset can support not only substantive analyses but also methodological work on retention, missingness, and intervention completion in athlete populations. Importantly, the analytic dataset is completer-based rather than based on imputed follow-up data. For secondary users, this makes it especially important to distinguish design-level counts from complete-case analysis files and to consider possible attrition-related bias in any downstream modeling.

Basic psychometric indicators suggest that the focal scales have generally strong internal consistency within this dataset. In the manuscript tables, the focal instruments show Cronbach's alpha values ranging from 0.76 to 0.97 and McDonald's omega values ranging from 0.77 to 0.97 across the two measurement waves. Sport persistence shows particularly high reliability, while the sport orientation subscales and grit subscales also demonstrate usable to strong internal consistency. These values make the dataset well suited for score-based secondary analysis, while the presence of multiple related constructs also supports factor-analytic, invariance-oriented, or network-based work, provided that users rely on the deposited item-level documentation.

A further strength of the dataset is its mixed intervention structure. Because the intervention arm was split into hybrid and online formats, and because the control arm remained untreated during the study period, the dataset can support several forms of secondary categorization: intervention vs. control comparisons, hybrid vs. online comparisons, and dose- or exposure-sensitive work if future documentation of completion intensity is linked to the public data package. Even when no confirmatory hypothesis testing is undertaken in the Data Report itself, these structural features increase the long-term value of the dataset for open secondary research.

Baseline descriptive statistics and between-group comparisons are presented in Tables 2, 3. Overall, the three study groups showed comparable baseline values on several psychological measures, including task orientation, sport persistence, perseverance of effort, wellbeing, future control, and concentration disruption, with no statistically significant differences observed. However, significant baseline differences were identified for several variables, including consistency of interest, resilience, positive future orientation, time management, future uncertainty, lack of self-efficacy, both rumination dimensions, and the somatic anxiety and negative thoughts subscales of sport anxiety. Effect sizes ranged from negligible to large (ε^2^ < 0.001–0.423), indicating that while some constructs were well balanced across groups, others exhibited meaningful baseline variability.

**Table 2:** Descriptive statistics of the study groups at baseline (pre-test).

Variable	Hybrid (*n* = 60) M ±SD	Online (*n* = 79) M ±SD	Control (*n* = 69) M ±SD
PSQ—task orientation	24.98 ± 6.15	25.43 ± 6.19	26.55 ± 4.15
PSQ—ego orientation	20.65 ± 6.32	18.61 ± 7.12	18.42 ± 6.30
SOQ—win orientation	21.30 ± 6.58	19.80 ± 7.20	19.65 ± 6.08
SOQ—goal orientation	14.68 ± 3.78	14.14 ± 3.95	13.93 ± 3.14
SOQ—competitiveness	11.27 ± 2.89	11.11 ± 3.05	11.23 ± 2.82
Sport persistence (SPQ)	49.25 ± 13.03	51.48 ± 13.81	51.67 ± 11.08
Grit—consistency of interest	12.43 ± 4.18	14.08 ± 3.74	11.17 ± 3.71
Grit—perseverance of effort	14.62 ± 3.85	14.63 ± 3.99	15.07 ± 3.26
Resilience^*^	33.11 ± 5.20	29.59 ± 8.04	8.88 ± 13.70
Wellbeing (WHO-5)	12.73 ± 5.40	13.19 ± 4.46	12.42 ± 4.55
Self in the future—positive future	13.37 ± 5.05	14.67 ± 4.67	12.64 ± 4.87
Self in the future—future control	13.03 ± 4.42	13.18 ± 3.72	12.42 ± 3.12
Self in the future—time management	12.05 ± 3.67	12.35 ± 3.70	9.97 ± 3.49
Self in the future—future uncertainty	14.47 ± 4.89	14.96 ± 5.11	11.35 ± 5.98
Self in the future—lack of self-efficacy	14.43 ± 3.89	15.11 ± 3.80	11.97 ± 3.11
Rumination—reflection^*^	7.74 ± 4.17	7.08 ± 4.20	1.83 ± 3.12
Rumination—brooding^*^	6.53 ± 3.56	7.18 ± 4.42	2.14 ± 3.61
Sport anxiety—somatic anxiety	9.75 ± 3.98	8.82 ± 3.73	12.30 ± 4.49
Sport anxiety—negative thoughts	12.53 ± 4.41	11.38 ± 4.74	15.59 ± 4.09
Sport anxiety—concentration disruption	9.55 ± 3.96	8.56 ± 3.43	9.42 ± 3.23

^*^means significant difference.

**Table 3:** Baseline (pre-test) differences between the three study groups (Kruskal–Wallis test).

Variable	*χ^2^*	df	*p*	*ε^2^*
PSQ—task orientation	0.855	2	0.652	0.004
PSQ—ego orientation	4.653	2	0.098	0.022
SOQ—win orientation	2.884	2	0.237	0.014
SOQ—goal orientation	3.111	2	0.211	0.015
SOQ—competitiveness	0.086	2	0.958	< 0.001
Sport persistence (SPQ)	2.399	2	0.301	0.012
Grit—consistency of interest	19.025	2	< 0.001	0.092
Grit—perseverance of effort	0.374	2	0.830	0.002
Resilience	70.266	2	< 0.001	0.423
Wellbeing (WHO-5)	1.252	2	0.535	0.006
Self in the future—positive future	6.767	2	0.034	0.033
Self in the future—future control	3.518	2	0.172	0.017
Self in the future—time management	15.449	2	< 0.001	0.075
Self in the future—future uncertainty	15.262	2	< 0.001	0.074
Self in the future—lack of self-efficacy	26.956	2	< 0.001	0.130
Rumination—Reflection	60.736	2	< 0.001	0.366
Rumination—Brooding	49.911	2	< 0.001	0.301
Sport anxiety—somatic anxiety	23.718	2	< 0.001	0.115
Sport anxiety—negative thoughts	30.659	2	< 0.001	0.148
Sport anxiety—concentration disruption	3.682	2	0.159	0.018

An attrition analysis was also conducted to compare participants who completed both measurement waves with those lost to follow-up. Baseline descriptive statistics and between-group comparisons are presented in Appendix Tables A1, A2. No statistically significant differences were observed between completers and non-completers on any of the baseline psychological measures (all *p* > 0.05). Moreover, effect sizes were uniformly negligible (ε^2^ ≤ 0.006), indicating that loss to follow-up was not systematically associated with the assessed baseline characteristics. These findings suggest that attrition was unlikely to introduce substantial bias into the final analytical sample and support the suitability of the complete-case dataset for secondary analyses.

## 4 How readers may interpret and reuse the dataset

The dataset is best interpreted as a structured youth-athlete intervention dataset with repeated psychological measurement and documented intervention delivery format. It should not be treated as a general population dataset, nor as a representative sample of all Hungarian athletes. Instead, it is most appropriate for questions concerning actively training student-athletes in adolescence and early adulthood. Its strongest reuse potential lies in psychometrics, intervention-methodology studies, attrition analyses, subgroup modeling, scale co-development work, and secondary theory testing on the relationships among persistence, motivation, anxiety, resilience, and wellbeing.

Because the source material involves human participants, reuse guidance should also specify anonymization boundaries. The public release should exclude direct or indirectly identifying information and should document any variables that were removed, generalized, or collapsed in order to preserve participant confidentiality. If any portion of the raw material cannot legally or ethically be shared, that limitation should be stated with precision. However, for a Data Report article, the openly reusable version must still be sufficient to justify the claim that a fixed public dataset has been deposited. In practical terms, this means that a request-only repository entry is not enough for this article type; a shareable anonymized release is needed.

## Data Availability

The datasets presented in this study can be found in online repositories. The names of the repository/repositories and accession number(s) can be found at: https://osf.io/h2zpg.

## References

[B1] Dombi E. (2018). Az időperspektíva hazai mérése, személyiségvonásokkal való összefüggéseinek vizsgálata és egy új jövőorientációs mérőeszköz kidolgozása.

[B2] Grugan M. C. Hill A. P. Mallinson-Howard S. H. Donachie T. C. Olsson L. F. Madigan D. J. . (2021). Development and initial validation of the perfectionistic climate questionnaire-sport (PCQ-S). Psychol. Sport Exerc. 56:101997. doi: 10.1016/j.psychsport.2021.101997

[B3] Gyömbér N. Kovács K. (2012). *Fejben dol el*. Sportpszichológia mindenkinek. Budapest: Noran Libro.

[B4] Hanrahan S. J. Cerin E. (2009). Gender, level of participation, and type of sport: differences in achievement goal orientation and attributional style. J. Sci. Med. Sport 12, 508–512. doi: 10.1016/j.jsams.2008.01.005 18356105

[B5] Hopewell S. Chan A.-W. Collins G. S. Hróbjartsson A. Moher D. Schulz K. F. . (2025). CONSORT 2025 statement: updated guideline for reporting randomised trials. BMJ 389:e081123. doi: 10.1136/bmj-2024-081123 40228833 PMC11995449

[B6] Ingrell J. Johnson U. Ivarsson A. (2020). Achievement goals in youth sport and the influence of coaches, peers, and parents: a longitudinal study. J. Hum. Sport Exerc. 15, 570–590. doi: 10.14198/jhse.2020.153.09

[B7] Járai R. Vajda D. László N. Hargitai R. Csókási K. Kiss E. C. (2015). A CONNOR-DAVIDSON REZILIENCIA KÉRDőÍV 10 ITEMES VÁLTOZATÁNAK JELLEMZőI. Alkalmazott Pszichológia 15, 129–136. doi: 10.17627/ALKPSZICH.2015.1.129

[B8] Kocsel N. Mónok K. Szabó E. Morgan A. Reinhardt M. Urbán R. . (2019). Nonproductive Thoughts Questionnaire for Children-Hungarian Version [Adatbázis]. doi: 10.1037/t74014-000 28459165

[B9] Kovács K. E. (2021). A sportperzisztencia vizsgálata az egészség, kapcsolati háló, motiváció és tanulmányi eredményesség függvényében. Iskolakultúra 31:5. doi: 10.14232/ISKKULT.2021.05.55

[B10] Kovács K. E. (2025a). Athlete profiles along grit, sport orientation and sport persistence based on a quantitative research on Hungarian athletes. Front. Sports Act. Living 7:1594365. doi: 10.3389/fspor.2025.1594365 40416053 PMC12098642

[B11] Kovács K. E. (2025b). Factors influencing sport persistence along the socio-ecological model—a presentation of sport persistence models based on the findings of a representative Hungarian sample. Sports 13:97. doi: 10.3390/sports13040097 40278723 PMC12030790

[B12] Kovács K. E. Csukonyi C. (2025). Investigating sport persistence through the development of the sport persistence questionnaire. Sci. Rep. 15:26049. doi: 10.1038/s41598-025-11231-3 40681541 PMC12274538

[B13] Kovács K. E. Kovács K. Szabó F. Dan B. A. Szakál Z. Moravecz M. . (2022). Sport motivation from the perspective of health, institutional embeddedness and academic persistence among higher educational students. Int. J. Environ. Res. Public Health 19:7423. doi: 10.3390/ijerph19127423 35742672 PMC9224480

[B14] Németh Á. (2023). Iskoláskorú gyermekek egészségmagatartása 2022. ELTE, Pedagógiai és Pszichológiai Kar. Available online at: https://hbsc.ppk.elte.hu/media/a3/fd/8127578da4d410a66e72f58fe74845e0cdfc9b890255362ba0e95ba4ca51/HBSC_2022_Riport.pdf (Accessed June 19, 2026).

[B15] Pikó B. Keresztes N. (2007). Sport, lélek, egészség. Dombi: Akadémiai Kiadó.

[B16] Roberts G. C. Treasure D. C. Balague G. (1998). Achievement goals in sport: the development and validation of the perception of success questionnaire. J. Sports Sci. 16, 337–347. doi: 10.1080/02640419808559362 9663958

[B17] Selejó Joó B. T. Czipa H. Bódi R. Lupócz Z. Paronai R. Tóth B. T. . (2024). Qualitative analysis of micro-system-level factors determining sport persistence. J. Funct. Morphol. Kinesiol. 9:196. doi: 10.3390/jfmk9040196 PMC11503412 39449490

[B18] Smith R. E. Smoll F. L. Cumming S. P. Grossbard J. R. (2006). Measurement of multidimensional sport performance anxiety in children and adults: the sport anxiety scale-2. J. Sport Exerc. Psychol. 28, 479–501. doi: 10.1123/jsep.28.4.479

[B19] Susánszky É. Thege B. Konkol. Stauder A. Kopp M. (2006). A WHO JÓL-LÉT KÉRDǑÍV RÖVIDÍTETT (WBI-5) MAGYAR VÁLTOZATÁNAK VALIDÁLÁSA A HUNGAROSTUDY 2002 ORSZÁGOS LAKOSSÁGI EGÉSZSÉGFELMÉRÉS ALAPJÁN. doi: 10.1556/mental.7.2006.3.8

[B20] Tóth B. T. Bódi R. Bodolai B. Ónadi Z. Kohut Z. Kovács K. E. (2026). The Role of social support and perfectionist climate in the development of sports persistence. Behav. Sci. 16:183. doi: 10.3390/bs16020183 41749992 PMC12938378

[B21] Tóth B. T. Tóth H. L. Lukácsi C. Kocsner O. C. Lovas B. Selejó Joó B. T. . (2024). Exploring individual components of sport persistence in the light of gender, education, and level and type of sport. J. Funct. Morphol. Kinesiol. 9:205. doi: 10.3390/jfmk9040205 39584858 PMC11587138

