# Supplementary material for: The sport persistence training dataset: a randomized controlled pre–post dataset on sport persistence and related psychological resources among young athletes in Hungary
Source: Front Psychol. 2026 Oct 8;17:1921608. doi: 10.3389/fpsyg.2026.1921608 (PMC13729011; doi:10.3389/fpsyg.2026.1921608)
Supplement: Supplementary file 1 [file Table_1.docx]

**Table A1.** Post-test differences between the three study groups (Kruskal–Wallis test)

| **Variable** | **Hybrid (n = 60) M ± SD** | **Online (n = 79) M ± SD** | **Control (n = 69) M ± SD** |
| --- | --- | --- | --- |
| PSQ – Task orientation | 27.73 ± 3.79 | 25.66 ± 5.30 | 25.04 ± 4.97 |
| PSQ – Ego orientation | 22.00 ± 5.46 | 21.66 ± 6.48 | 21.19 ± 5.66 |
| SOQ – Win orientation | 22.32 ± 5.69 | 23.03 ± 5.66 | 22.35 ± 5.39 |
| SOQ – Goal orientation | 17.70 ± 3.35 | 17.25 ± 3.25 | 16.46 ± 3.35 |
| SOQ – Competitiveness | 12.45 ± 2.45 | 12.05 ± 2.42 | 10.90 ± 3.02 |
| Sport Persistence (SPQ) | 55.33 ± 11.12 | 51.63 ± 12.49 | 48.03 ± 14.61 |
| Grit – Consistency of interest | 9.10 ± 4.45 | 12.22 ± 4.20 | 12.86 ± 3.90 |
| Grit – Perseverance of effort | 16.63 ± 2.99 | 15.04 ± 3.73 | 14.17 ± 3.83 |
| Resilience* | 29.05 ± 8.63 | 30.68 ± 7.99 | 28.17 ± 8.60 |
| Well-being (WHO-5) | 15.55 ± 3.70 | 13.44 ± 4.18 | 12.67 ± 3.93 |
| Self in the Future – Positive future | 15.88 ± 3.76 | 15.59 ± 4.20 | 14.23 ± 4.77 |
| Self in the Future – Future control | 13.88 ± 3.17 | 13.47 ± 3.07 | 13.25 ± 4.41 |
| Self in the Future – Time management | 10.82 ± 3.06 | 13.24 ± 4.00 | 11.20 ± 3.27 |
| Self in the Future – Future uncertainty | 7.88 ± 4.80 | 14.44 ± 4.86 | 13.52 ± 5.44 |
| Self in the Future – Lack of self-efficacy | 10.60 ± 2.62 | 15.18 ± 3.84 | 13.90 ± 3.73 |
| Rumination – Reflection* | 8.00 ± 4.41 | 8.78 ± 4.04 | 7.59 ± 4.07 |
| Rumination – Brooding* | 8.00 ± 4.41 | 8.78 ± 4.04 | 7.59 ± 4.07 |
| Sport Anxiety – Somatic anxiety | 10.48 ± 3.77 | 9.51 ± 3.97 | 10.70 ± 3.86 |
| Sport Anxiety – Negative thoughts | 12.73 ± 4.17 | 12.28 ± 4.75 | 12.51 ± 4.19 |
| Sport Anxiety – Concentration disruption | 7.98 ± 4.14 | 9.28 ± 4.09 | 9.49 ± 3.42 |

**Table A2.** Post-test differences between the three study groups (Kruskal–Wallis test)

| **Variable** | **χ²** | **df** | **p** | **ε²** |
| --- | --- | --- | --- | --- |
| PSQ – Task orientation | 16.591 | 2 | < .001 | .080 |
| PSQ – Ego orientation | 0.497 | 2 | .780 | .002 |
| SOQ – Win orientation | 1.087 | 2 | .581 | .005 |
| SOQ – Goal orientation | 5.706 | 2 | .058 | .028 |
| SOQ – Competitiveness | 11.111 | 2 | .004 | .054 |
| Sport Persistence (SPQ) | 11.217 | 2 | .004 | .054 |
| Grit – Consistency of interest | 27.130 | 2 | < .001 | .131 |
| Grit – Perseverance of effort | 15.536 | 2 | < .001 | .075 |
| Resilience | 4.737 | 2 | .094 | .029 |
| Well-being (WHO-5) | 18.766 | 2 | < .001 | .091 |
| Self in the Future – Positive future | 4.210 | 2 | .122 | .020 |
| Self in the Future – Future control | 1.754 | 2 | .416 | .008 |
| Self in the Future – Time management | 15.421 | 2 | < .001 | .075 |
| Self in the Future – Future uncertainty | 47.157 | 2 | < .001 | .228 |
| Self in the Future – Lack of self-efficacy | 50.773 | 2 | < .001 | .245 |
| Rumination – Reflection | 2.441 | 2 | .295 | .015 |
| Rumination – Brooding | 2.441 | 2 | .295 | .015 |
| Sport Anxiety – Somatic anxiety | 4.583 | 2 | .101 | .022 |
| Sport Anxiety – Negative thoughts | 0.255 | 2 | .880 | .001 |
| Sport Anxiety – Concentration disruption | 11.109 | 2 | .004 | .054 |
